# Glutathione S-Transferase Polymorphism Interactions with Smoking Status and HPV Infection in Cervical Cancer Risk: An Evidence-Based Meta-Analysis

**DOI:** 10.1371/journal.pone.0083497

**Published:** 2013-12-31

**Authors:** Shuai Zhen, Chen-Ming Hu, Li-Hong Bian

**Affiliations:** 1 Xijing Hospital, Fourth Military Medical University, Xi'an, China; 2 Department of Gynecology, The 307 Hospital of the Academy of Military Medical Sciences, Beijing, China; University of North Carolina School of Medicine, United States of America

## Abstract

**Background:**

Human papillomavirus (HPV) infection is considered the major cause of cervical cancer (CC), but a number of infected women do not develop invasive lesions, suggesting the role of genetic susceptibility and environmental co-factors for cancer outbreak. Glutathione S- transferases (GSTs) are multifunctional enzymes that play a key role in the detoxification of varieties of both endogenous products of oxidative stress and exogenous carcinogens.

**Methods:**

MEDLINE, EMBASE, and Cochrane databases were searched. All studies evaluating the association between GSTM1 polymorphisms and cervical cancer were included. Pooled odds ratio (OR) and 95% confidence interval (CI) were calculated using fixed-or random-effects model.

**Results:**

A total of 23 case-control studies were included in the meta-analysis. The overall result showed that the association between GSTM1 null genotype and risk for cervical cancer was statistically significant (OR = 1.56; 95%CI, 1.39–1.75). Subgroup analyses were performed based on ethnicity, smoking and HPV infection. Our results showed that smokers with null GSTM1 genotype had higher risk of cervical cancer (OR = 2.27, 95%CI, 1.46–3.54). For the ethnicity stratification, significant increased risk of null GSTM1 genotype was found in Chinese and Indian population, but no increased risk in other population was found.

**Conclusions:**

this meta-analysis provided strong evidence that the GSTM1 genotype is associated with CC development, especially in Chinese and Indian populations. Smoking and HPV infection modified the association between the null GSTM1 genotype and CC.

## Introduction

Cervical cancer (CC) is the second most common gynecologic malignancy in the world and the seventh most frequent overall malignancy [1]. Generally, more than 85% of the global burden occurs in developing countries, where CC accounts for 15% of all female cancers. High-risk regions include Eastern and Western Africa, Southern Africa, South America, and Middle Africa. The incidence rates are the lowest in Western Asia and Northern America. The different incidence rates in different areas indicate that genetic and environmental factors play roles in CC development.

According to several epidemiological and biological studies, human papilloma virus (HPV) infection is the dominant etiological event in CC development; however, it is insufficient as a causal agent because this virus is also detected at a certain frequency among women who are cytologically normal, and CC occurs in only a fraction of HPV-infected women. Additional features of the host, including an active sexual history, weakened immune function, and cigarette smoking, have been confirmed as risk factors for CC [2]. Among these factors, smoking is associated with a significantly increased risk, and its effects, which are enhanced by interaction with HPV infection [3] but appear to be independent of socioeconomic status and sexual behavior [4], are dose-dependent [3]. In fact, cigarette smoke carcinogens, polycyclic aromatic hydrocarbons (PAHs), and benzo(α)pyrene have been detected in the cervical mucus of smokers, while cigarette smoke carcinogen-specific DNA adducts (e.g. NNK) have been found in the cervical epithelial cells of cigarette smokers [5]. These results suggest that tobacco smoking could increase the risk of tumor onset and viral infection persistence. Therefore, molecular studies have identified polymorphic gene products that are associated with tobacco smoke procarcinogen metabolism and thus might determine the individual predisposition to CC.

Previous studies have shown that genetic variations in the glutathione S-transferases (GSTs) affect human phase II detoxification enzymes involved in the detoxification of various exogenous and endogenous reactive species [6]. Cytosolic GSTs play a role in the conjugation of glutathione to the products of endogenous lipid peroxidation and detoxification of tobacco smoke-associated carcinogenic aflatoxin electrophiles and PAHs. The mode of action of GSTs is thought to involve simultaneous enzyme activation and detoxification and GSTs could affect the modulation of reactive species that form DNA adducts and cause somatic mutations [7]. Accordingly, several studies have identified an association between genetic polymorphisms of GSTs and the risk of cancer development.

GSTM1 facilitates the excretion of a wide range of carcinogens, reactive oxygen species, and chemotherapeutic agents with a variety of substrate specificities. Its allelic variant *0 (null allele) caus7es a completely lack of enzymatic activity to bind genotoxic substrates such as epoxides derived from aflatoxin and PAHs [8]. Many epidemiological studies have evaluated GSTM1 and the risk of CC in different populations; however, the results have been inconsistent [9], [10]. Although a few meta-analyses regarding gene polymorphisms and CC have been performed, to our knowledge, no gene–environment interactions have been explored, especially regarding HPV. We wanted to investigate whether some GST polymorphisms could influence the risk of CC development in a cohort of HPV-infected women, either alone or in combination with a smoking habit. Therefore, we conducted a meta-analysis regarding the effect of GSTM1 gene polymorphisms on the CC risk and explored the gene–environment interaction with regard to CC risk.

## Materials and Methods

This meta-analysis was conducted in accordance with the Preferred Reporting Items for Systematic Reviews and Meta-analyses (PRISMA) guidelines [11]. A pre-specified protocol that included the data sources, search strategy, inclusion/exclusion criteria for the articles, and analysis methods was developed before the beginning of this study.

### Selection criteria and search strategy

**Figure 1:**
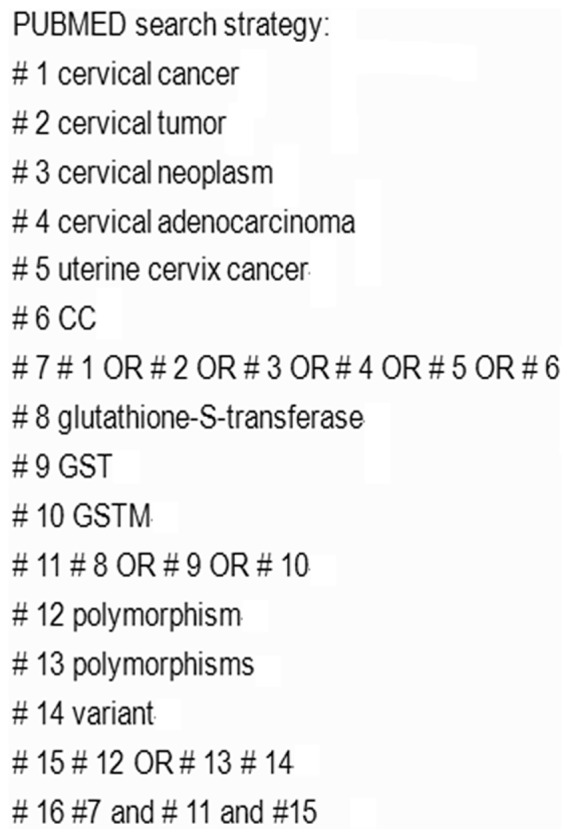
PUBMED search strategy.

We followed the Meta-analysis Of Observational Studies in Epidemiology (MOOSE) [12]. A systematic search was conducted by the authors (Z.S. and H.C.M). The identification of relevant studies was conducted in a search of the Cochrane Databases, PubMed, Medline, and EMBASE up to June 2013, using the following terms without any restriction on language. The search terms were “cervical cancer”, “cervical tumor”, “cervical neoplasm”, “cervical adenocarcinoma”, “uterine cervix cancer”, “CC”, “glutathione-S-transferase”, “GST”, “GSTM”, “polymorphism”, “polymorphisms”, and “variant”. The PubMed search strategy is shown in (Figure 1). The references of all eligible articles were checked for other relevant articles.

### Inclusion/exclusion criteria

The inclusion criteria were as follows: case-control studies that investigated the association between GSTM1 polymorphisms and the risk of CC and studies that presented original data and the numbers of null GSTM1 genotypes among the cases and controls.

Exclusion criteria included the following: duplicate publications; case reports; insufficient data to construct a 2×2 table of the test result; precancerous lesions included among the cases; lack of a control group; and abstracts, reviews, talks, and review class documentations.

### Data extraction and quality assessment

The data extracted from each study included the authors, publication year, country of origin, average ages of cases and controls, number of null GSTM1 genotypes among the cases and controls, and the adjusted ORs of the selected studies. Smokers were defined as those with a lifetime history of smoking more than 100 cigarettes and who had smoked continuously for at least 1 year until the time of diagnosis; non-smokers were defined as those who had never smoked. The 2×2 tables were reconstructed. Two (Z.S. and H.C.M.) authors independently assessed the articles according to the inclusion/exclusion criteria and referred to Corresponding Author (B.L.H.) in cases of disagreement. When important data were not provided, the authors contacted the article authors via e-mail.

The study quality was assessed according to the revised tool for the quality assessment of diagnostic accuracy studies (QUADAS-2; a description is shown in Table 1) [13] and the standards for reporting diagnostic accuracy (STARD) tool [14]. Each item was scored as a “yes”, “no”, or “unclear” if there was insufficient information to make an accurate judgment.

**Table 1:** QUADAS List.

Item No.	Description
1	Representative patient spectrum
2	Clear description of selection criteria
3	Acceptable reference standard
4	Acceptable delay between tests
5	Avoiding partial verification bias
6	Sufficient differential verification bias
7	Avoiding incorporation bias
8	Sufficient description of index test
9	Sufficient description of reference test
10	Blinded interpretation of index test results
11	Blinded interpretation of index reference results
12	Availability of clinical data to the researchers
13	Reporting of uninterpretable indeterminate results
14	Explanation of withdrawals from study

### Statistical analysis

Data analysis was performed using Review Manager (version 5.0) and STATA software (version 11, StataCorp LP, College Station, TX, USA).The association between the GSTM1 gene polymorphisms and CC was estimated by calculating the pooled odds ratios (ORs) with 95% confidence intervals (CIs). ORs were used to analyze the results, and their corresponding 95% CIs were estimated. Cross-study heterogeneity was estimated using the I^2^-statistic and Q-statistic [15]. The meta-analysis was conducted using the random-effects or fixed effects methods model, based on the pooled effect estimates in the presence (p<0.1 and I^2^>50%) or absence (p>0.1 and I^2^<50%) of heterogeneity [16]. An evaluation of the potential publication bias was estimated by constructing funnel plots for visual inspection and Egger's regression asymmetry test [17]. Studies were categorized into subgroups based on ethnicity, HPV infection, and smoking status.

## Results

### Characteristics and quality assessment of the included studies

**Figure 2:**
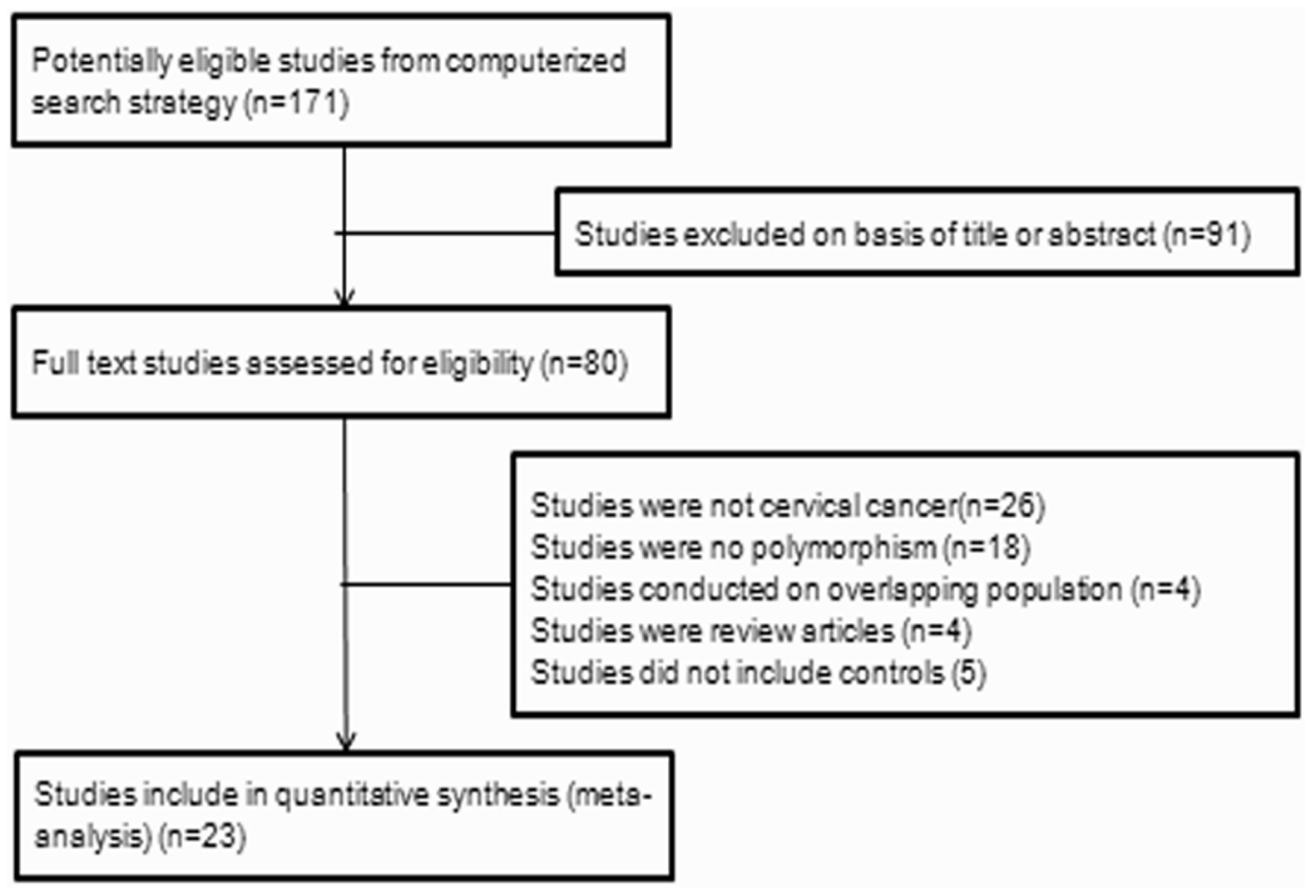
Flow diagram of study selection.

**Figure 3:**
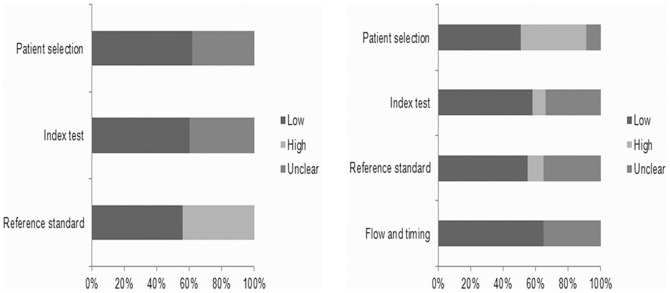
Graphical display of study characteristics according to QUADAS II recommendations. (Left: Proportion of studies with low, or high, or unclear concerns regarding applicability; Right: Proportion of studies with low, high or unclear risk of bias.).

A flow diagram of the study selection process is shown in Figure 2. The literature search identified 171 potentially relevant studies; of these, 91 were excluded after screening the titles and abstracts. The full-text studies were retrieved for a detailed assessment. Fifty-seven were excluded for various reasons (26 studies did not involve CC, 18did not involve polymorphisms, 5 did not include controls, 4 were conducted on overlapping populations, and 4 were review articles). Finally, 23 case-control studies [18], [19], [20], [21], [22], [23], [24], [25], [26], [27], [28], [29], [30], [31], [32], [33], [34], [35], [36], [37], [38], [39], [40] were included in the GSTM1 genotype meta-analysis (2343 cases and 2662 controls). The meta-analysis included studies from China, India, Japan, Korea, Italy, USA, Greece, Brazil, Turkey, and Thailand. The characteristics of studies included in the meta-analysis are presented in Table 2. The results of the QUADAS-2 assessment of the included studies are shown in Figure 3.

**Table 2:** Characteristics of studies included in the meta-analysis.

Study	Year	Ethnicity	Study design	Mean age of cases	Mean age of controls	Cases/Controls	Null GSTM1 genotype Cases/Controls
Agodi [18]	2010	Italy	Hospital	NA	NA	27/162	15/17
Agorastos [19]	2007	Greece	Hospital	NA	NA	176/114	33/60
de Carvalho [20]	2008	Brazil	Hospital	NA	NA	43/86	28/49
Goodman [21]	2001	America	Population	32.3	39.1	131/180	74/98
Huang [22]	2006	China	Hospital	40.8	41.2	47/78	30/32
Joseph [23]	2006	India	Population	46	47	147/165	79/54
Kim [24]	2000	Korea	Population	46.5	46.5	181/181	95/96
Kiran B [25]	2010	Turkey	Hospital	NA	NA	46/52	15/16
Lee [26]	2004	Korea	Hospital	NA	NA	81/86	42/42
Liu [27]	2009	China	Hospital	NA	NA	62/45	40/13
Ma [28]	2009	China	Hospital	46.7	48.8	43/45	29/15
Nishino [29]	2008	Japan	Population	41.6	40.6	124/125	77/59
Niwa [30]	2005	Japan	Hospital	47.2	56.2	131/320	70/184
Palma [31]	2010	Italy	Hospital	NA	NA	81/111	49/58
Seltheetham-Ishida [32]	2009	Thailand	Population	NA	NA	69/72	54/56
Sharma [33]	2004	India	Hospital	NA	NA	142/96	81/33
Sierra-Torres [34]	2003	America	Population	38.3	34.8	69/72	35/29
Sierra-Torres [35]	2006	America	Population	45.5	42.3	91/92	36/38
Singh [36]	2008	India	Population	45.2	50.3	150/168	64/40
Sobti [37]	2006	India	Hospital	48.6	48	103/103	42/38
Song [38]	2006	China	Hospital	49.1	47.2	130/130	77/57
Ueda [39]	2008	Japan	Population	NA	NA	144/54	75/28
Zhou [40]	2006	China	Hospital	40.7	50.5	125/125	73/54

### Data synthesis and meta-analysis

**Figure 4:**
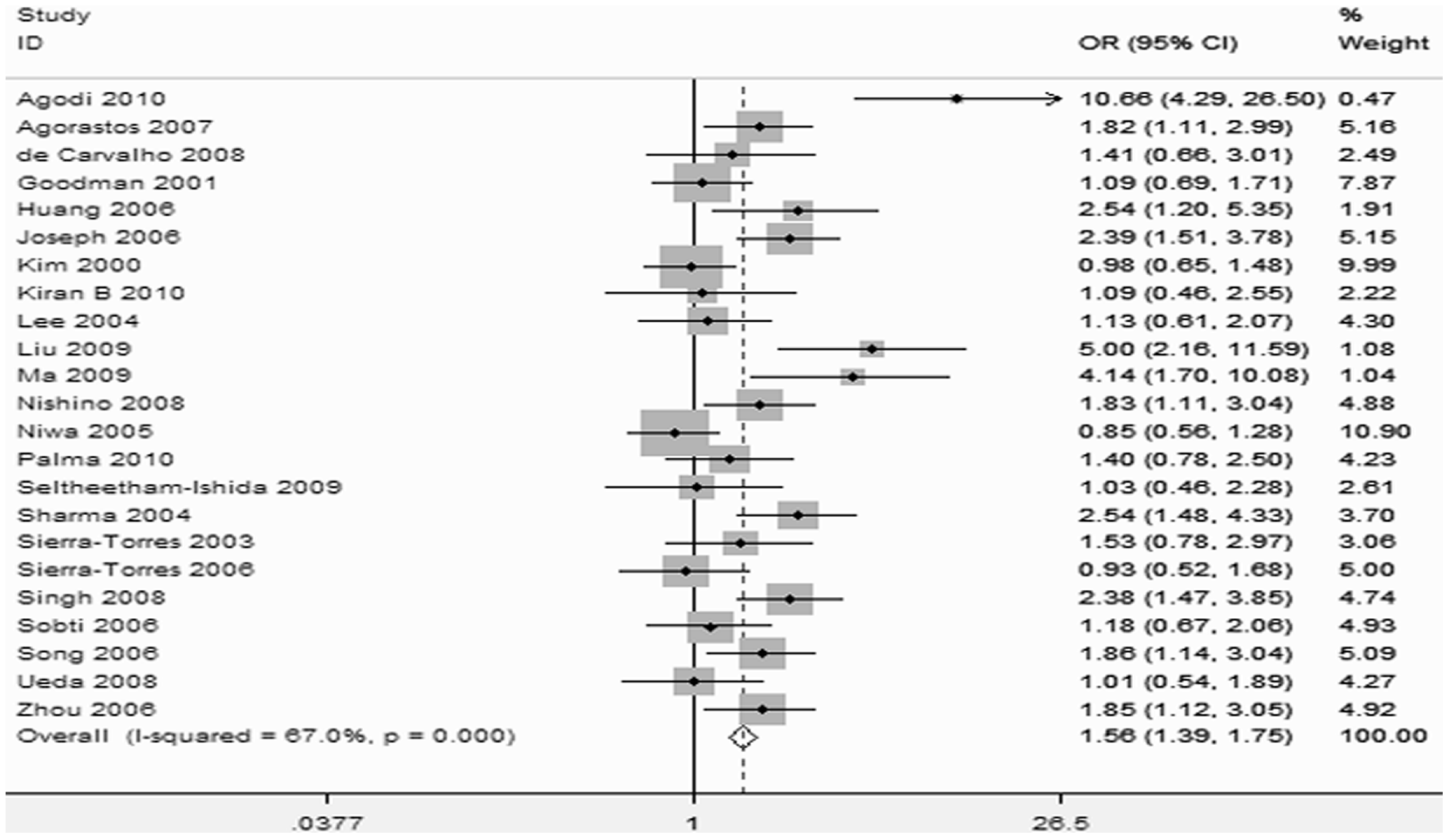
Forest Plot for the Overall Association Between GSTM1 Gene Polymorphism and Cervical Cancer Risk.

The forest plot of the GSTM1 meta-analysis is shown in Figure 4. Heterogeneity was observed in the GSTM1 studies (p<0.001, I^2^ = 67% for GSTM1), and therefore, a random-effects model was used. The overall result showed that the GSTM1 null allele was related to an increased risk of CC (OR = 1.56; 95%CI, 1.39–1.75).

**Figure 5:**
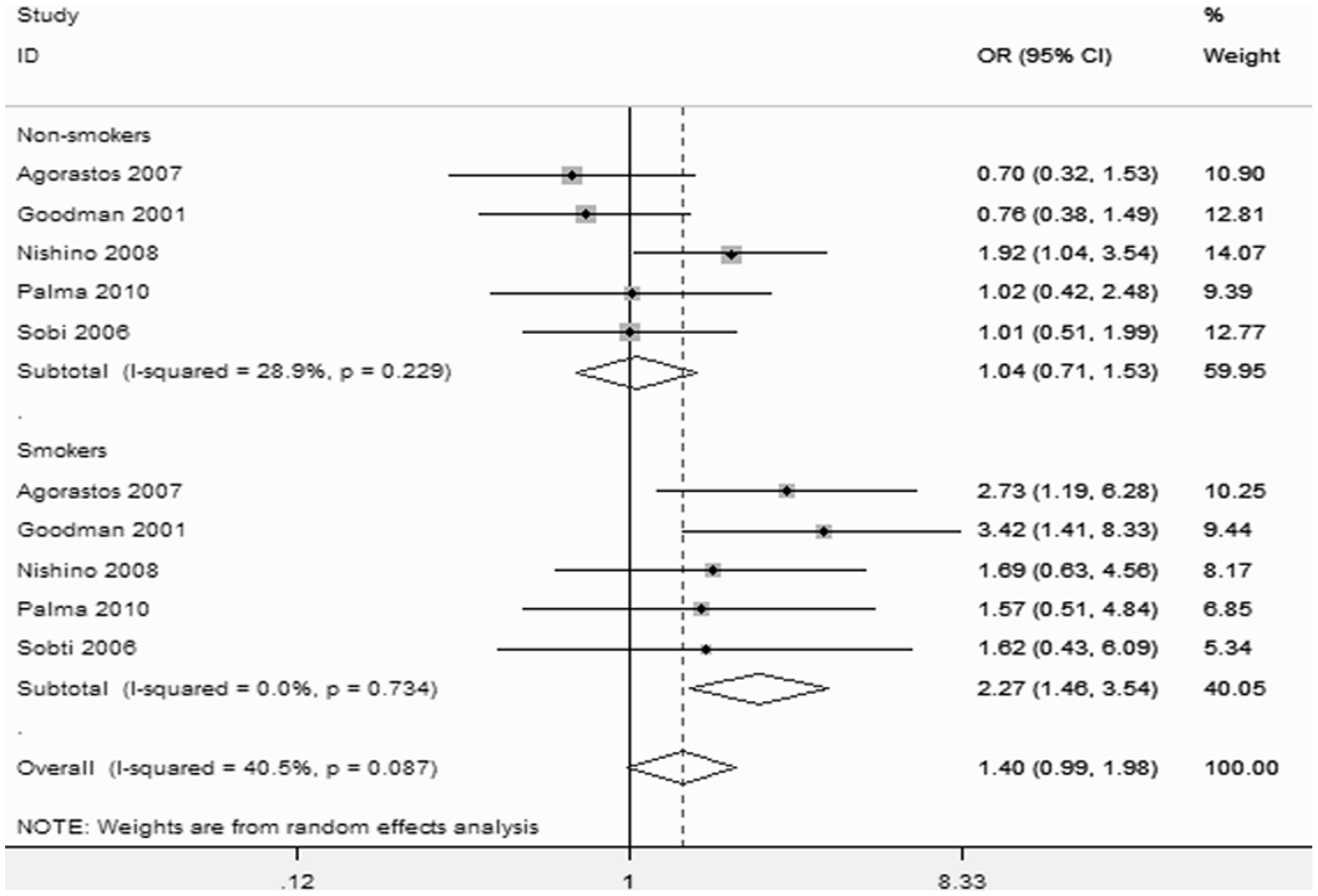
Relationship Between GSTM1 Gene Polymorphism and Cervical Cancer Risk by Smoking Status.

**Figure 6:**
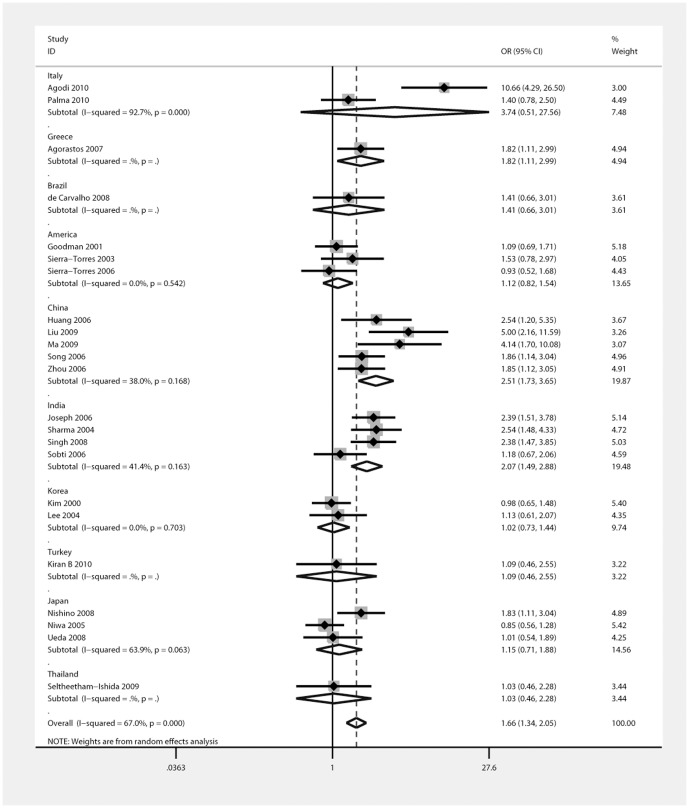
Relationship Between GSTM1 Gene Polymorphism and Cervical Cancer Risk by Ethnicity.

**Figure 7:**
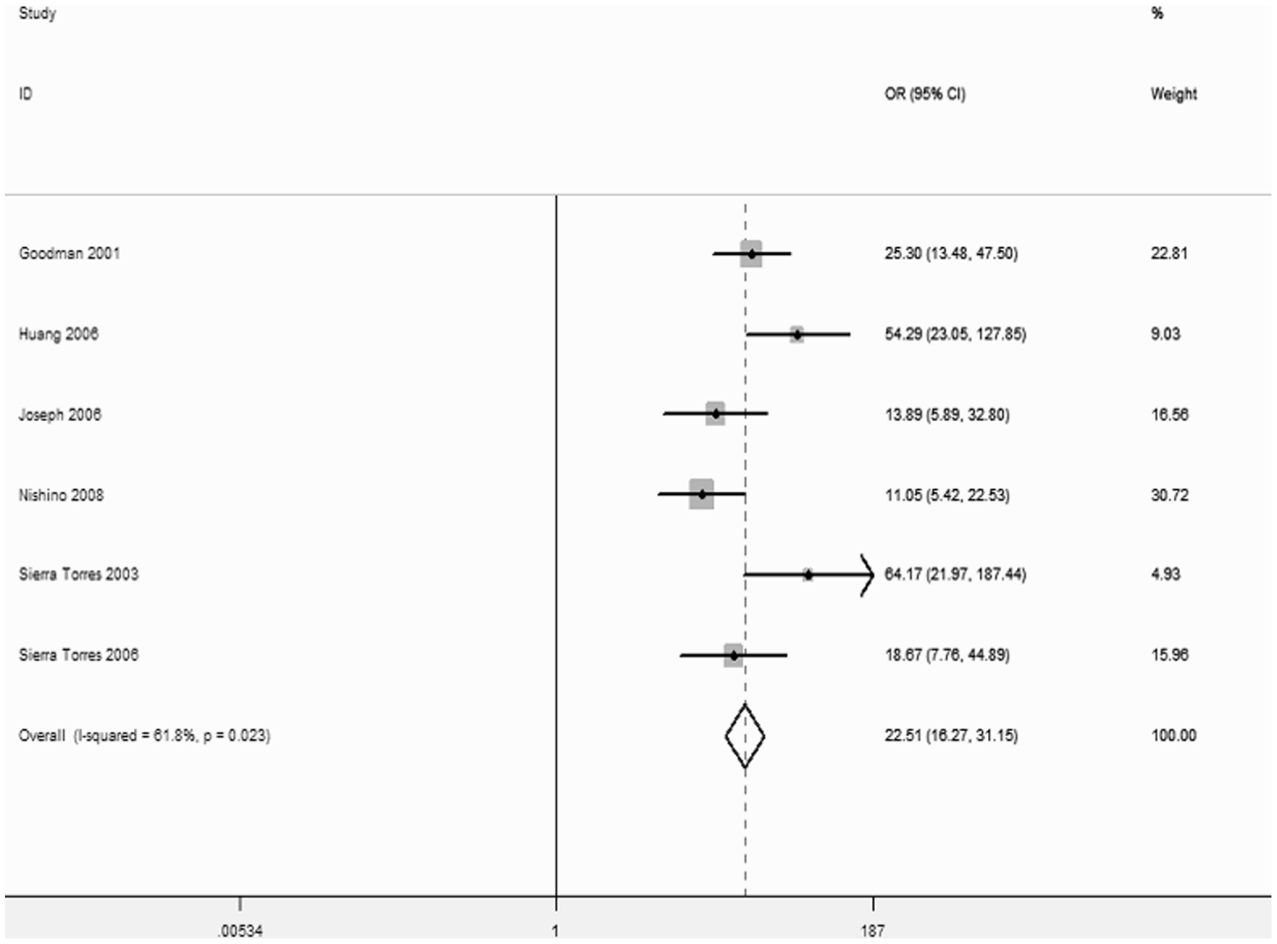
Relationship Between GSTM1 Gene Polymorphism and Cervical Cancer Risk by HPV infection.

Subgroup analyses were performed according to smoking history, ethnicity, and HPV infection status. The results showed that smokers had an increased risk of CC (OR = 2.27; 95%CI, 1.46–3.54), while no significant increased risk was observed in non-smokers (Figure 5). After stratification, the heterogeneity decreased significantly (P = 0.229, I^2^ = 28.9% for non-smokers and P = 0.734, I^2^ = 0% for smokers). In the ethnicity stratification, a moderately significant increase in risk was associated with the null GSTM1 genotype in Chinese (OR = 2.51; 95%CI, 1.73–3.65) and Indian populations (OR = 2.07; 95%CI, 1.49–2.88), but the risk observed in other populations was not significant (Figure 6). In the HPV infection status stratification, the results showed that HPV infection was associated with the risk of CC (OR = 22.51; 95%CI, 16.27–31.15; I^2^ = 61.8%, P = 0.023; Figure 7).

### Heterogeneity assessment

Significant cross-study heterogeneity was present in both the overall and subgroup analyses. We explored several possible sources of this cross-study heterogeneity, such as ethnicity, the DNA genotyping sample source, sample size, and quality score. However, none of these variables could explain the heterogeneity, which could have been caused by the limited number of included studies. Regardless of the p value, ethnicity and study design were thought to play important roles. Next, we filtered 3 studies (American population and study design are population-based) and obtained an I^2^of 0%. Further studies are needed to confirm the roles of ethnicity and study design with regard to heterogeneity.

## Discussion

Despite CC being among the most common cancers worldwide, the etiology is not yet clearly understood. The present and related studies show that the GST-null genotype is associated with an increased cancer incidence. Moreover, GSTs also play an important modulatory role in the induction of other enzymes and proteins involved in cellular functions such as DNA repair [41]. The relationship between GST gene polymorphisms and CC has been investigated in various studies. However, the association between these variables has been controversial, and discrepancies could have been due to limited sample numbers and ethnic differences. Our meta-analysis showed a possible role for GSTM1 polymorphism, which interacts with smoking status and HPV infection status, in the promotion of CC development; also the risk of CC was statistically significant in Chinese and Indian populations, but not in other population, indicating that these differences in cancer susceptibility varied according to ethnicity. Additionally, the data showed that the frequency of the GSTM1 null allele genotype was higher in American and Japanese populations than in Chinese and Indian populations, suggesting that the varied effects of the genotype might be attributable to differences in lifestyle, nutrition, environmental factors, and genetic factors.

Cigarette smoking, which decreases the ability to clear oncogenic infections, has been found to be an independent risk factor of CC [42]. Jee et al. [43] reported that ever smokers with the GSTP1 A/A (variant allele, homozygous) genotype had an increased risk of invasive CC. Because most women in this region rarely smoke, the exposure to smoke is passive; this type of exposure has been found to increase the risk of CC. A significant association was found between CC and passive smoking, but not active smoking [44].Our study showed that tobacco components are modified by metabolic enzymes and can promote malignant cellular growth. The mode of action occurs through the activation and detoxification of tobacco carcinogens; therefore, GST polymorphisms might be expected to affect the risk of cancer development among smokers. The absence of GST activity, which is caused by an inherited deletion of the GST, has been reported to increase the risk of several tobacco-related cancers. We therefore hypothesized that smoking and the GST genotype might synergistically influence CC development. Our study showed that the null GSTM1 genotype might increase the CC risk among smokers, thus providing strong evidence for an association between GSTs and CC risk.

In previous decades, a few meta-analyses that investigated the association between GST polymorphisms and CC found that this association is, however, affected by an important limitation [45]. For example, the study failed to perform a subgroup analysis according to major environmental determinations such as the HPV infection status.

Our meta-analysis included some limitations. First, heterogeneity was a major problem in this meta-analysis. We explored several possible sources of heterogeneity, including the sample size and DNA source. However, we failed to find a clear reason, indicating that a conservative manner should be adopted when interpreting these results. Second, some control sources were population-based [46], while others were hospital-based; the latter are more prone to bias than are the former. Finally, the sample size reported in the literature is still relatively small and might not provide sufficient power to estimate the association between the null GSTM1 polymorphism and CC risk.

In conclusion, this meta-analysis provided strong evidence that the GSTM1 genotype is associated with CC development, especially in Chinese and Indian populations. Smoking and HPV infection modified the association between the null GSTM1 genotype and CC. Studies with large sample sizes should be performed to confirm this finding. Additionally, further studies that investigate the effects of gene–environment interactions on CC risk are required.

## Supporting Information

Checklist S1 **PRISMA checklist.**
(PDF)

Checklist S2 **PRISMA Flow Diagram.**
(PDF)

## References

[1] Parkin DM, Bray F, Ferlay J, Pisani P (2005) Global cancer statistics, 2002. CA Cancer J Clin 74–108. 15761078 10.3322/canjclin.55.2.74

[2] Zeller JL, Lynm C, Glass RM (2007) JAMA patient page (2007) Carcinoma of the cervix. JAMA 298: 2336. 18029838 10.1001/jama.298.19.2336

[3] Kjellberg L, Hallmans G, Ahren AM, Johansson R, Bergman F, et al (2000) Smoking, diet, pregnancy and oral contraceptive use as risk factors for cervical intra-epithelial neoplasia in relation to human papillomavirus infection. Br J Cancer 82: 1332–1228. 10755410 10.1054/bjoc.1999.1100 PMC2374476

[4] Slattery ML, Robison LM, Schuman KL, French TK, Abbott TM, et al (1989) Cigarette smoking and exposure to passive smoke are risk factors for cervical cancer. JAMA 261: 1593–1598. 2918652

[5] Prokopczyk B, Cox JE, Hoffmann D, Waggoner SE (1997) Identification of tobacco-specific carcinogen in the cervical mucus of smokers and non-smokers. J Natl Cancer Inst 89: 868–73. 9196253 10.1093/jnci/89.12.868

[6] Hengstler JG, Arand M, Herrero ME, Oesch F (1998) Polymorphisms of N-acetytransferases, glutathione S-transferases, microsomal epoxide hydrolase and sulfotransferases: influence on cancer susceptibility. Recent Results Cancer Res 154: 47–85. 10026993 10.1007/978-3-642-46870-4_4

[7] Miller MC, Mohrenweiser HW, Bell DA (2001) Genetic variability in susceptibility and response to toxicants. Toxicol Lett 120: 269–80. 11323185 10.1016/s0378-4274(01)00279-x

[8] Hayes JD, Pulford DJ (1995) The glutathione S-transferase supergene family: regulation of GST and the contribution of the isoenzymes to cancer chemoprotection and drug resistance. Crit Rev Biochem Mol Biol 30: 445–600. 8770536 10.3109/10409239509083491

[9] Agodi A, Barchitta M, Cipresso R, Marzagalli R, La Rosa N, et al (2010) Distribution of p53, GST, and MTHFR polymorphisms and risk of cervical intraepithelial lesions in sicily. Int J Gynecol Cancer 20 1: 141–146. 20130515 10.1111/IGC.0b013e3181c20842

[10] Agorastos T, Papadopoulos N, Lambropoulos AF, Chrisafi S, Mikos T, et al (2007) Glutathione-S-transferase M1 and T1 and cytochrome P1A1 genetic polymorphisms and susceptibility to cervical intraepithelial neoplasia in Greek women. Eur J Cancer Prev 16: 498–504. 18090121 10.1097/01.cej.0000243859.99265.92

[11] Moher D, Liberati A, Tetzlaff J, Altman DG (2009) the PRISMA group (2009) Preferred reporting items for systematic reviews and meta-analyses: the PRISMA statement. J Clin Epidemiol 62: 1006–1012. 19631508 10.1016/j.jclinepi.2009.06.005

[12] Stroup DF, Berlin JA, Morton SC, Olkin I, Williamson GD, et al (2000) Meta-analysis of observational studies in epidemiology: a proposal for reporting. Meta-analysis Of Observational Studies in Epidemiology (MOOSE) group. JAMA 283: 2008–2012. 10789670 10.1001/jama.283.15.2008

[13] Whiting PF, Rutjes AW, Westwood ME, Mallett S, Deeks JJ, et al (2011) QUADAS-2: a revised tool for the quality assessment of diagnostic accuracy studies. Ann Intern Med 155: 529–536. 22007046 10.7326/0003-4819-155-8-201110180-00009

[14] Bossuyt PM, Reitsma JB, Bruns DE, Gatsonis CA, Glasziou PP, et al (2003) Towards complete and accurate reporting of studies of diagnostic accuracy: the STARD initiative. Standards for Reporting of Diagnostic Accuracy. Clin Chem 49: 1–6. 12507953 10.1373/49.1.1

[15] Higgins JP, Thompson SG (2002) Quantifying heterogeneity in a meta-analysis. Stat Med 21: 1539–1558. 12111919 10.1002/sim.1186

[16] DerSimonian R, Laird N (1986) Meta-analysis in clinical trials. Control Clin Trials 7: 177–188. 3802833 10.1016/0197-2456(86)90046-2

[17] Egger M, Davey Smith G, Schneider M, Minder C (1997) Bias in meta-analysis detected by a simple, graphical test. BMJ 315: 629–634. 9310563 10.1136/bmj.315.7109.629 PMC2127453

[18] Agodi A, Barchitta M, Cipresso R, Marzagalli R, La Rosa N, et al (2010) Distribution of p53, GST, and MTHFR polymorphisms and risk of cervical intraepithelial lesions in sicily. International Journal of Gynecological Cancer 20: 141–146. 20130515 10.1111/IGC.0b013e3181c20842

[19] Agorastos T, Papadopoulos N, Lambropoulos AF, Chrisafi S, Mikos T, et al (2007) Glutathione-S-transferase M1 and T1 and cytochrome P1A1 genetic polymorphisms and susceptibility to cervical intraepithelial neoplasia in Greek women. Eur J Cancer Prev 16: 498–504. 18090121 10.1097/01.cej.0000243859.99265.92

[20] De Carvalho CR, da Silva ID, Pereira JS, de Souza NC, Focchi GR, et al (2008) Polymorphisms of p53, GSTM1 and GSTT1, and HPV in uterine cervix adenocarcinoma. Eur J Gynaecol Oncol 29: 590–593. 19115684

[21] Goodman MT, McDuffie K, Hernandez B, Bertram CC, Wilkens LR, et al (2001) CYP1A1, GSTM1, and GSTT1 polymorphisms and the risk of cervical squamous intraepithelial lesions in multiethnic population. Gynecol Oncol 81: 263–269. 11330960 10.1006/gyno.2001.6154

[22] Huang YK, Hsieh HC, Sun JA, Chao CF, Huang RL, et al (2006) Genetic polymorphisms of phase I and phase II xenobiotic enzymes in human papillomavirus related lesion and cancer of the uterine cervix. Tzu Chi Medical Journal 18: 267-27+328.

[23] Joseph T, Chacko P, Wesley R, Jayaprakash PG, James FV, et al (2006) Germline genetic polymorphisms of CYP1A1, GSTM1 and GSTT1 genes in indian cervical cancer: associations with tumor progression, age and human papillomavirus infection. Gynecol Oncol 101: 411–417. 16360200 10.1016/j.ygyno.2005.10.033

[24] Kim JW, Lee CG, Park YG, Kim KS, Kim IK, et al (2000) Combined analysis of germline polymorphisms of p53, GSTM1, GSTT1, CYP1A1, and CYP2E1: relation to the incidence rate of cervical carcinoma. Cancer 88: 2082–2091. 10813720 10.1002/(sici)1097-0142(20000501)88:9<2082::aid-cncr14>3.0.co;2-d

[25] Kiran B, Karkucak M, Ozan H, Yakut T, Ozerkan K, et al (2010) GST (GSTM1, GSTT1, and GSTP1) polymorphisms in the genetic susceptibility of Turkish patients to cervical cancer. J Gynecol Oncol 21: 169–73. 20922139 10.3802/jgo.2010.21.3.169 PMC2948224

[26] Lee SA, Kim JW, Roh JW, Choi JY, Lee KM, et al (2004) Genetic polymorphisms of GSTM1, p21, p53 and HPV infection with cervical cancer in Korean women. Gynecol Oncol 93: 14–18. 15047208 10.1016/j.ygyno.2003.11.045

[27] Liu Y, Ma W, Liu Q, Yang T, Xu X, et al (2009) Association between genetic polymorphism of GSTM1, CYP2E1 and susceptibility to cervical cancer and its precancerous lesions in Uighur women in Xinjiang. Prog Obstet Gynecol 18: 840–843.

[28] Ma C, Liu X, Ma Z (2009) Association between genetic polymorphism of GSTM1 and susceptibility to cervical cancer in Uighur women in Xinjiang. Chin J Obstet Gynecol 44: 629–631.

[29] Nishino K, Sekine M, Kodama S, Sudo N, Aoki Y, et al (2008) Cigarette smoking and glutathione S-transferase M1 polymorphism associated with risk for uterine cervical cancer. J Obstet Gynaecol Res 34: 994–1001. 19012698 10.1111/j.1447-0756.2008.00798.x

[30] Niwa Y, Hirose K, Nakanishi T, Nawa A, Kuzuya K, et al (2005) Association of the MAD(P)H: quinone oxidoreductase C609T polymorphism and the risk of cervical cancer in Janpanese subjects. Gynecol Oncol 96: 423–429. 15661231 10.1016/j.ygyno.2004.10.015

[31] Palma S, Novelli F, Padua L, Venuti A, Prignano G, et al (2010) Interaction between glutathione-S-transferase polymorphisms, smoking habit, and HPV infection in cervical cancer risk. J Cancer Res Clin Oncol 136: 1101–9. 20069434 10.1007/s00432-009-0757-3 PMC11827813

[32] Settheetham-Ishida W, Yuenyao P, Kularbkaew C, Settheetham D, Ishida T (2009) Glutathione S-transferase (GSTM1 and GSTT1) polymorphisms in cervical cancer in Northeastern Thailand. Asian Pac J Cancer Prev 10: 365–368. 19640174

[33] Sharma A, Sharma JK, Murthy NS, Mitra AB (2004) Polymorphisms at GSTM1 and GSTT1 gene loci and susceptibility to cervical cancer in Indian population. Neoplasma 51: 12–6. 15004652

[34] Sierra-Torres CH, Au WW, Arrastia CD, Cajas-Salazar N, Robazetti SC, et al (2003) Polymorphisms for chemical metabolizing genes and risk for cervical neoplasia. Environmental and Molecular Mutagenesis 41: 69–76. 12552594 10.1002/em.10132

[35] Sierra-Torres CH, Arboleda-Morreno YY, Orejuela-Aristizabal L (2006) Exposure to wood smoke, HPV infection, and genetic susceptibility for cervical neoplasia among women in Colombia,. Environ Mol Mutagen 47: 553–561. 16795085 10.1002/em.20228

[36] Singh H, Sachan R, Devi S, Pandey SN, Mittal B (2008) Association of GSTM1, GSTT1, and GSTM3 gene polymorphisms and susceptibility to cervical cancer in a North Indian population. Am J Obstet Gynecol 198: 303e301–306. 18177825 10.1016/j.ajog.2007.09.046

[37] Sobti RC, Kaur S, Kaur P, Singh J, Gupta I, et al (2006) Interaction of passive smoking with GST (GSTM1, GSTT1, and GSTP1) genotypes in the risk of cervical cancer in India. Cancer Genet Cytogenet 166: 117–123. 16631467 10.1016/j.cancergencyto.2005.10.001

[38] Song GY, Song ZY, Xu JP, Shao SL (2008) Association of single nucleotide polymorphism in glutathione S-transferase-M1 with susceptibility to cervical cancer in Shanxi Province. Chinese Journal of Cancer Prevention and Treatment 15: 1054–1056.

[39] Ueda M, Toji E, Nunobiki O, Izuma S, Okamoto Y, et al (2008) Germline polymorphism of cancer susceptibility genes in gynecologic cancer. Human Cell 21: 95–104. 19067761 10.1111/j.1749-0774.2008.00058.x

[40] Zhou Q, Wang J, Shao S, Ma X, Ding L (2006) The association between glutathione S-transferase M1, T1 polymorphisms and risk of cervical cancer. Modern Preventive Medicine 33: 269–271.

[41] Der Simonian R, Laird N (1986) Meta-analysis in clinical trials. Control Clin Trials 7: 177–88. 3802833 10.1016/0197-2456(86)90046-2

[42] Giuliano AR, Sedjo RL, Roe DJ, Harris R, Baldwin S, et al (2002) Clearance of oncogenic human papilloma virus (HPV) infection: effect of smoking (United States). Cancer Causes Control 13: 839–46. 12462549 10.1023/a:1020668232219

[43] Jee SH, Lee JE, Kin S, Kim JH, Um SJ, et al (2002) GSTP1 polymorphisms, cigarette smoking and cervical cancer risk in Korean women. Yonsei Med J 6: 712–6. 10.3349/ymj.2002.43.6.712 12497653

[44] Trimble CL, Genkinger JM, Burke AE, Hoffman SC, Helzlsouer KJ, et al (2005) Active and passive cigarette smoking and risk of cervical neoplasia. Obstet Gynecol 105: 174–81. 15625160 10.1097/01.AOG.0000148268.43584.03 PMC3064987

[45] Ying Liu, Liang-Zhi Xu (2012) Meta-analysis of association between GSTM1 gene polymorphism and cervical cancer. Asian Pacific Journal of Tropical Medicine 480–484. 22575983 10.1016/S1995-7645(12)60083-2

[46] Wacholder S, Silverman DT, Mclaughlin JK, Mandel JS (1992) Selection of controls in case-control studies II. Types of controls. Am J Epidemiol 135: 1029–41. 1595689 10.1093/oxfordjournals.aje.a116397

